# The Bandage in the Treatment of Fractures, Ulcers, Gun-Shot Wounds, Hemorrhage, &c., &c.

**Published:** 1850-05

**Authors:** Theodore S. Bell

**Affiliations:** Louisville, Ky.


ART. II.—*The Bandage in the Treatment of Fractures, Ulcers, Gun-Shot Wounds, Hemorrhage, &c., &c.* By THEODORE S. BELL, M. D., of Louisville, Ky.

There is scarcely any class of diseased actions, that are consigned to the care of the surgeon, that receives a greater variety of treatment, than that which passes under the name of ulcers. Amidst the bewildering lights of authorities, on the method of treatment, it is difficult for the tyro to make a proper selection, and when he passes beyond the primeval stage of his surgical life, he soon, as a general rule, degenerates into the practices of the routinist. Authorities generally tell us to make special applications to the local affection, and these are usually so much in unison with the patient's ideas of the proper method of treatment that they are readily resorted to. Balsam of Peru, the terebinthinates, citric unguents, solution of sulphate of copper, fumigations of sulphur and iodine, blistering ointments, aconitum napellus, black wash, creosote, electricity, iodate of the protoxide of mercury, iodide of starch, oxide of silver, pectic acid as a poultice, periodic acid, the bromates, succon-

---

ates, mucic acid, alloxan, hydrated peroxide of iron, are a portion of the things that are extravagantly lauded in modern practice, and they constitute a wilderness of perplexity that must puzzle any one in attempting to travel through it. In the chase after these false lights, the philosophy of ulceration, and the proper treatment of the diseased actions are lost sight of.

In a former number of this Journal, an attempt was made to call the attention of the profession to the testimony of distinguished authorities in surgery, to the uses of the bandage in fractures, gun-shot wounds, and ulcers. There was, of course, no thought of setting up a claim to originality in the views advanced. In the full blaze of the lights reflected from the pages of Heister's "General System of Surgery," Underwood's "Treatise on Ulcers of the Legs," Baynton's "Descriptive Account of a New Method of Treating Old Ulcers of the Legs," Whately's "Practical Observations on the Cure of Wounds and Ulcers on the Legs, without rest," and John Bell's "Principles of Surgery," from the latter of which extended quotations were made in a former paper on the bandage, all bearing testimony to the great utility of the roller, in the treatment of the surgery indicated in these remarks, such a claim would be in the highest degree preposterous, and the man who should make it, would show such a contemptuous appreciation of the intellect of his readers, that he would be justly amenable to the scorn of the profession. No modern authority has, in my judgment, improved upon the practical views of Heister and John Bell, upon the various uses of the bandage, and the highest efforts of a reader of the works of these surgeons, must be made by enforcing their views, by calling attention to their valuable lessons, and in restoring to them their just and honest fame.

In view of these facts, and of the neglect of the authorities I have named, I quote from Mr. Critchett's lectures on "Ulcers of the Lower Extremities," delivered in the London Hospital," in the summer of 1848, the fol lowing just and appropriate views:

"There exists such a plethora in medical literature, that it behooves each new commentator upon Nature's morbid operations, if he would expect to obtain any attention from his professional brethren, to accomplish one of three objects;—he must either propound new and original views, founded on legitimate induction, or he must collect the rays of light that lie scattered over the fields of pathology, and present them in a clear, concentrated and useful form; or, lastly, he must resuscitate some old principle, valuable in a practical point of view, which, from some accidental cause, has been allowed to slumber in neglect and oblivion; it is in this latter and humbler capacity that the author of these lectures breaks silence; he claims not the high honor of having discovered a new window, whereby light may be admitted into our surgical temple, but simply to have rendered transparent one that had become obscured and useless. The author's main object, then, in the publication of this little book, is to place the principle of mechanical support, in the treatment of ulcers of the lower limbs, on a correct and scientific basis; for although, as he has elsewhere endeavor ed to show, this has already been briefly done by the late Mr. John Scott, in his work 'On Diseased Joints,' a reference to all the best surgical authorities since that work appeared, proves how entirely that principle is still, if not unknown, at least most imperfectly expounded."

These are the sentiments I feel on the subject of the bandage, and the views by which I desire to be judged. I am aiming at "the old paths" of surgery, because I believe them to be infinitely more useful, more practical, more philosophic and successful than the most of the new ones in the modern school. I am convinced that the ancient lights of the profession are too much neglected in the race after the "new things" of the day. In our ignorance of what the ancient observers have recorded, we are often deceived by claims of originality in things that are as old as the time of Hippocrates. A medical writer, of some celebrity, pointed out a number of instances of this kind a few years ago, and his references to the observations of Areteus, and Galen, may surprize some of our readers. The doctrine of the distinct function of the sensatory and motor nerves, over which modern physiologists are growling, and the proof of which doctrine conferred so much deserved fame on Sir Charles Bell, was alluded to by Areteus, and "distinctly and emphatically propounded by Galen." We have not space to spare for a quotation from Galen, but the doctrine is lucidly set forth by him in the ninth and fourteenth chapters of his fifth book: "De usu Partium." Philosophical Anatomy is considered quite a modern science, but Aristotle was almost as deeply imbued with it as the Meckels and Serres of our day. Cuvier labored in what he considered original investigations, but when he came to study the four books of Aristotle, "De Partibus Animalium," he was surprized and delighted to find that the comprehensive and acute mind of the Stagyrite had forestalled him in a great variety of the principal facts of philosophical anatomy.

The writer to whom I have alluded, says: "That view of life which regards it, whether in its healthy or its morbid manifestations, as a state of perpetual relation to the agents by which the living organism is surrounded, has resulted from a very advanced stage of physiological and pathological knowledge, after the successive explosion of a variety of hypotheses." Yet, this is precisely the view that was taken by Hippocrates, and we have no doubt of a fact that has been stated in connection with this subject, that Hippocrates "cultivated medicine on more enlarged and scientific principles than most of those who call themselves medical reasoners, even in the present day."

Again: the crude condition of chemistry in the days of Hippocrates and Galen, afforded them no aid in their prelections on the fluids of the human body. They constructed a fantastic system of humoralism, which should have been improved by the lights of an advancing chemistry. But, instead of doing that, as has been correctly said, "modern inquirers ran into an extreme solidism, as false, in point of fact, as, and infinitely more absurd in reasoning than the old humoral doctrines, the insufficiency of which was demonstrated by the progress of chemical science." If, instead of running after the vagaries of solidism, with its myriad of errors, "men had been content with rectifying the humoralism of the ancients, by the facts of modern chemistry, we should, by this time, have attained to views of organic chemistry which might have been brought into very profitable relations with medicine, instead of being, as we are, on the mere threshold of the subject."

The same writer says: " Not only, then, the germs, but a pretty ample development of all those doctrines which have given the greatest impulse to medicine in our own times, are to be found in the writings of the ancients."—In view of these facts, the readers of these remarks will rejoice to learn, that the French Academy is preparing an edition of the works of the "Fathers of Medicine," and that they intend to make it, in every way, worthy of the Academy. Neither expense, time, labor, nor investigation will be spared in making a library of ancient medical literature that will be invaluable to the medical student.

These things have been referred to, in order to show the readers of these papers the value of the "old paths" which were opened in the first paper on the bandage, and which it is my design now to pursue again. Wiseman,

Baynton, and Scott gained no small degree of credit by their use of the bandage, but Heister preceded them in all the useful qualities of that agent. I should rejoice to see the following sentiment of old Hiester properly impressed upon the mind of every person who wishes to practice surgery beneficially to his patients, and creditably to himself. That surgical reputation that is founded upon a thorough and correct appreciation of the bandage, cannot be easily shaken. Heister said, in 1739:* "The general use and necessity of bandages in relieving and curing the disorders of human bodies, is very apparent, not only from their being thought worthy to be made an important subject of consideration by the first fathers of physic, as Hippocrates and Galen, with other eminent physicians; but also from there being hardly any operation in surgery practicable without their assistance. Even where an operation has been performed, in all other respects, with the greatest judgment and dexterity, yet if the surgeon miscarry in his bandage, by an unskilful application thereof, all his other endeavors, though just and laudable, may either totally, or in a great measure, prove fruitless, to the great damage of his reputation; and this more especially in the treatment of wounds, fractures, luxations, amputations, and the like. We may add, that in fractures, and luxations, after a reduction of the parts, the whole cure depends entirely on the bandage, and, in many profuse hemorrhages, nothing can afford so certain and speedy relief, as an exact deligation of the wound with a fit compress and bandage, which may even save the life of the patient, as every one knows that has the least knowledge of the nature and treatment of wounds. To say nothing of the recommendation, that the neatness and readiness of making a bandage and dressing will give the surgeon, both as to his patient and the spectators,

* Heister's Institutions of Surgery. Part third, p. 292.

who judge of his other abilities by his performance of what comes under the general cognizance of every one's senses, as Galen justly observes."*

I know of no more important paragraph in surgery than the one just quoted from Heister, and all who may acquire proper facilities and understanding in the use of the bandage will, after the experience of a few years has taught them its value, fully corroborate the remark made above. Heister employed the bandage quite as extensively as any modern surgeon does, and for the same objects. His name is sometimes appealed to as an authority for the use of balsam of Peru for ulcers, but an examination of his remarks scarcely supports the idea that he was favorable to any thing of the kind. He says: "It is not easy to say what should induce almost all physicians to cry up certain balsamic remedies, as having a peculiar virtue in generating new flesh: besides, our digestive is endowed with a balsamic power; *but to say the truth, the generation of new flesh is not so much owing to the use of any particular medicines, as to the benefit of Nature.*"

I have made these references to the ancient literature of surgery and medicine, in the hope of inducing some readers to look into the works of the founders of medicine. I am persuaded that much has been lost by neglecting their writings, and that practical medicine would now be much farther advanced, if they had been treated differently. Of one thing the reader may rest assured—his examination of these ancient writers will satisfy him that many things which are claimed as new, among moderns, were well known and appreciated by the early observers of medicine. Many a modern writer has claimed for himself an overwhelming originality in matters that were perfectly familiar to the profession centuries ago, and these

---

* Lib. de Fasciis, where he directs: Quod injicitur, celeriter, jucunde, prompte et eleganter injiciatur.

pseudo-originals gather around them a clan, who vindicate the former existence of Balaam's beast, by showing that they are his lineal descendants. Their actions, in relation to their master, perpetually repeat the language of their ancestor: "Am I not thine ass, upon which thou hast ridden ever since I was thine ass?"

It would be needless to urge the importance of correct views of the pathology and treatment of ulcerative action. The student will hasten from almost anything to see the operation of lithotomy, and in the course of years of practice he may never be called on to see a case of calculous disease; but his introduction into practice may probably be based upon the treatment of ulcers, and upon his success must depend his standing. Should he not, then devote himself to that which is most likely to pave the way to his professional eminence? Sir Benjamin Brodie, quoted by Mr. Critchett, has expressed the sentiments that should govern all medical men in matters of this nature. That distinguished surgeon, who had attained the highest distinctions of his profession, from the exalted position he had reached, thus showers down the ripe fruits of a long and well matured experience: "Ulcers of the leg are cases in which there is no question about the patient's life or death; and I think it very probable that many among you may pass by the bedside of such a patient, without thinking it worthy of attention. But I am not disposed to regard it in this manner. Although the patient may not die of this malady, yet without care it may render him miserable for life. The disease may be very much relieved by proper treatment, and it is one of very common occurrence. You examine carefully a case of aneurism, a case of stone in the bladder, and so on; but these are things of comparatively rare occurrence, and which will not fall under your treatment in the beginning of your professional lives; but ulcers of the leg are cases of a very distressing nature, and such as meet you at every turn of your practice; and your reputation in early life will depend more upon your understanding a case of this kind than upon your knowledge of one of more rare occurrence."

There are few diseased actions that are of more interest than those developed in the various forms of ulcers of the lower extremities. It is of the greatest importance that their character shall be well understood, and that a successful treatment shall be inculcated. An indication has been given, in the first of this paper, of the dire confusion that prevails among surgical writers on this subject. It is distressing even to look at an inventory of the articles commended for the cure of ulcers, and the fact that we often meet with descriptions of "old ulcers" is conclusive proof how futile and fruitless the articles of this inventory are in their application to ulcerative inflammation.

I do not purpose to urge the bandage as the sole agent for the treatment of ulcers; my design is to show what an important element it is in the treatment of those affections, and to point out the cases that call for its agency, and the conditions that require other means in aid of its powers. I have repeatedly seen an ample trial given to the various topical applications that have been enumerated in the commencement of these remarks, and have enjoyed abundant opportunities for comparing the results of the roller treatment, with every other form of management that has been commended, and I feel no kind of misgiving as to the truth of the statement, that the comparison was equal to the contrast between light and darkness, between uniform success and uniform failure, between comfort, ease, rapid amendment, complete and permanent recovery, and pain, restlessness, long continued suffering, and utter failure. It is humiliating to take up such a work as Sir Everard Home's Treatise on Ulcers, to read it, in full view of the amazing opportunities he enjoyed for attaining full knowledge of the whole philosophy of ulcerative action, and its proper management, and then turn to the lights of experience and observation. I am almost tempted to wish that Sir Everard Home had burned his own work on ulcers, rather than the immense masses of John Hunter's manuscripts, which he destroyed, to keep the world from discovering that he owed all his honors, his reputation, and distinction to his pilferings from his immortal kinsman.

In order to facilitate a full and correct understanding of the subject to be submitted to the reader, I shall divide this paper into departments. The first of these will properly be:

*The Causes of Ulceration.*—A full understanding of the laws of the venous circulation, will pave the way to a correct understanding of the phenomena of ulceration, especially in the lower extremities. Nine ulcers in ten occur in the lower extremities, and the most intractable forms are generally in the vicinity of the malleoli. These facts are constantly insisted upon by surgical authorities; the varicose condition of the saphena veins is usually connected with ulceration, and this condition was fully recognized by Heister, and by numerous authors since his time. John Hunter's investigations threw a great deal of light upon the process of ulceration, and his school of surgeons have made great improvements in the treatment.

Sir Benjamin Brodie, in his clinical lectures on surgery, has done the subject more than ordinary justice.

Mr. Critchett, in a series of lectures delivered in the London Hospital, has added valuable principles to the treatment of ulceration.

The two surgeons last named, insist with much force upon the varicose condition of the veins, in connection with the generality of ulcerations of the lower extremities. In addition to this, the locality of an ulcer has much to do with its entire character. In the language of Mr. Critchett, "when ulcers occur in the lower extremity, the more remote the position of the sore from the center of circulation, *(cæteris paribus,)* the more tedious and uncertain is the reparative process." This fact should admonish the surgeon of the necessity of increasing the energies of his efforts at reparative measures in a ratio with the remoteness of the ulcer from the center of circulation. Another important feature in the treatment, arising from the position of the ulcer, will be insisted upon in another part of this paper.]

Mr. Critchett insists upon the fact that cramps in the muscles of the legs have something to do with a varicose condition of the veins—a statement more plausible in theory than in reality. Mr. C. argues that in cramps of the legs, a pressure is made upon the deep-seated veins, and thus a larger amount than the usual quantity of the returning blood is thrown into the superficial veins, which causes dilatation. In this way the valves are separated, and, when their uses are gone, they shrivel, and, in a great measure, perish. This theory is scarcely borne out by observation. I have known many persons who were subject to repeated attacks of cramps in the legs, for years, who were not more subject to ulcerations than those who were not the victims of cramps. Cholera, in which cramps of the legs are of a violent character, has not increased the tendency to ulcerations, and children who are peculiarly liable to spasms, are not peculiarly the subjects of ulcerations.

Of the value of the following deductions of Mr. Critchett, there can be no kind of doubt, and they are important to a correct uuderstanding of the subject of ulceration. Mr. Critchett says: "A dilated condition of the veins of the lower extremity, from whatever cause it may spring, may terminate in five different ways:

"First. It may produce hypertrophy of the coats of the veins, and thus resist farther distention.

"Secondly. The principal veins may become plugged up more or less completely with fibrine, and thus by diverting the current of blood into other channels, the disease may become stationary.

"Thirdly. The capillaries of the skin and subcutaneous cellular tissue may become likewise dilated, giving rise to permanent discoloration of a large portion of the skin, and to thickening and firm fibrinous deposit beneath it, and thus the disease in the larger trunks would seem to be checked, if not arrested.

"Fourthly. The larger veins may become more and more enlarged and elongated; the smaller trunks may become gradually implicated, until what I have denominated the spongy leg is produced; this condition sometimes relieves itself by hemorrhage.

"Fifthly. In this spongy condition of leg a congeries of red vessels may form, in the centre of which a white patch appears on the skin, which is the immediate forerunner and indicator of ulceration; which, being established, is another way in which the congested veins are temporarily relieved."

As Mr. Critchett very properly remarks, this state of things is not only a frequent cause of ulcers, but also a serious barrier to their successful treatment, and a very constant source of their recurrence.

One of the most common causes of this varicose condition of the veins of the legs in females, is the pressure of the gravid uterus, and I have often seen alarming hemorrhages in this condition. Avocations that require persons to stand a long time in one position, is another fruitful source of it. The most common cause, in the cases that have come under my observation, is debilitating attacks of fever, which, by enfeebling the vital forces of the veins, produce engorgements in the superficial veins of the lower extremities. That form of fever, called the "Cold Plague," which raged, many years ago, through the United States, might have been tracked, from one end of the Union to the other, by the frightful and disastrous ulcerations it caused. It made a saturnalia for manufacturers of wooden legs.

A plethoric habit may easily cause ulcerations; the carbuncle may be considered a form of ulceration, and that is often the result of free eating. The use of alcoholic drinks is a well known cause of a very intractable kind of ulcer. There are few that are more difficult to manage, and none that are more apt to return. Those who have had anything to do with these ulcers, are fully cognizant of the difficulties that attend their permanent cure.

Deranged menstruation gives origin, sometimes, to what is called the menstrual ulcer, and as the treatment is modified by the character of its origin, it should be clearly recognized. In deciding upon the treatment of any ulcer, it is important that the cause of the diseased action shall be accurately known.

*Division of Ulcers.*—A great many distinctions have been enumerated in classifying ulcers, but the best classification I have seen is the one proposed by Mr. Critchett. He divides ulcers into simple, or local; and specific, or constitutional.

The simple, or local, are divided into acute, or spreading, subacute, chronic, healthy, irritable and varicose.—The specific, or constitutional, are arranged under the various heads of strumous, syphilitic, phagedenic, periosteal, menstrual, edematous, and malignant.

It is well to bear in mind these distinctions, for the proper treatment of these diseased actions depends mainly upon the ability to recognize the precise characters of each one. Upon a full knowledge of the laws of the venous, absorbent, and secreting systems, a nice discrimination of the varying phases of ulcerative action, and a clear and satisfactory diagnosis in the varieties of ulcers, mainly depends the success of the treatment. It must be ever borne in mind that what may be serviceable at one stage of an ulcer, may be injurious in a different stage.

I do not think it necessary to encumber this paper with a minute description of the ulcers already enumerated.—A brief sketch of a few of the prominent ulcers will be sufficient for the purposes of this essay.

*The Acute Ulcer.*—There is no scarcity of descriptive accounts of ulcers, but the authority I prefer is Mr. Critchett. His descriptions most nearly accord with my own experience and observation. Of the acute ulcer, he says: "This form of ulcer is attended generally with severe suffering, which is greatly aggravated when the limb is placed in a depending position. There is great heat and tension, and the pain is of an aching, growing character. The ulcer looks uneven and glassy, the edges are irregular and undefined, sloughs, varying in size, are often seen on the surface; the discharge is thin and ichorous; the surrounding parts are of a bright red color, or sometimes of a peculiar speckly red and white aspect." Mr. Critchett objects, it seems to me strangely, to the use of the bandage in this stage, but he very properly urges the use of the water dressing. I know of no application of the bandage more uniformly useful than in this form of ulcer. In combination with the water dressing, there is nothing more to be desired.

In the *sub-acute stage* of ulceration, the points to be attended to, are the color and temperature of the adjacent parts, and Mr. Critchett adds, as a useful guide, the amount of pressure required to empty the vessels, and the rapidity with which they re-fill.

*The Chronic Ulcer.*—This is the form of ulcer most generally presented to the notice of the physician. The acute and sub-acute stages are usually treated with mullen leaves and poultices of urine, and, proving intractable under such scientific assaults, they pass into the hands of the surgeon. The leading characteristics are, almost an absence of heat, and a discoloration of the skin, running into a blue or russet color. The sore looks as if it were deep; the edges may be everted, or inverted, or the face of the ulcer may present a flat appearance. These peculiar edgings often give occasion for the display of meddlesome scalpels and scissors.

*The Irritable Ulcer.*—This ulcer, according to Mr. Critchett, is usually small and superficial; is most commonly found in females; is generally situated about the ankle; and sometimes, but not invariably, complicated with varicose veins. The distinguishing characteristic is that the appearance and symptoms give no adequate suggestion of the extreme amount of tenderness and suffering experienced by the patient.

The specific ulcers, the strumous, phagedenic, and syphilitic, it is unnecessary to describe with anything like a detail of differences. Their character can be easily detected, and the treatment will be indicated in its proper department. I pass to a general survey of the subject.

If it were not my desire to make this essay practical in its character, I should be tempted to give a historical sketch of the treatment of ulcers, and the commendations of the bandage, as the chief agent of their cure, from the earliest era of medicine down to the present time. It is remarkable that in all epochs of surgery, traceable by written records, the bandage, as used in the present day, has been uniformly commended by able, judicious, and experienced men, and has been disregarded by the mass of practitioners. It is so much easier work, on the part of the surgeon, to order a poultice, a wash, or an unguent, than to apply a bandage neatly and properly, that this valuable agent does not enjoy that position, generally, to which it is entitled. The general ignorance, indeed, is so great, of the character of its merits, that men who have urged its value with zeal and earnestness, in the very terms and extensive appropriations given it by various authors, at least one hundred years since, have been very foolishly looked upon as original. If there is any use of the bandage taught now, that was not forcibly commended by Heister, Heberden, Wiseman, Underwood, Benjamin, and John Bell, to say nothing of many others, I confess I do not know anything about it. To men well read in professional literature, these modern claims to originality are in the highest degree preposterous, absurd and ridiculous. It is recorded of the editor of a daily newspaper, yet in existence, that he once had the fifth chapter of Matthew's gospel imposed upon him as an original communication, and that was scarcely more absurd than the ridiculous originality to which these remarks refer.

In order to impress this subject, treated in my former essay on the bandage, upon the mind of the reader, and to pave the way to a proper understanding of the treatment of ulcers, which I design to commend, I present the following quotations from various authors on the use and value of the bandage:

Heister gives directions for discharging matter from ulcers, etc., and directs to dress with "digestive ointment spread upon lint, and secured upon the part with diachylon diapalma, or any other plaster of that kind, covering the whole with proper compresses and bandages." Gen. Sys. Surg., part i., p. 242.

"*Fistulous Cases.*"—"When the ulcer is cleansed, and the proper dressings applied, you must clap a small compress, or a slip of plaster doubled up in the form of a small compress, upon the part where you judge the fundus of the fistula to be seated, securing all with bolster, plaster, and bandage, as usual. In rolling up, the best method will be to place the beginning of the roller upon the fundus of the fistula, or at least to make the fastening tight upon that part: This will direct the contained matter towards the opening, and the bottom will heal before the rest of the sinus." Ibid., p. 244.

Heister lays great stress on the importance of constitutional treatment, and says: "If internal remedies are not neglected, there is no doubt but that the very worst of ulcers may generally be cured." Ibid., p. 246.

"The surgeon's principal care in fractures, is to unite the broken bone, to which three things are necessary: 1. That the bone be restored to its natural situation, which is to be done by extending it and replacing it. 2. That after the bone has recovered its natural situation, it be kept there by giving it rest, and applying proper bandages. Lastly, 3. You are to use proper means to prevent, or remedy the disorders that usually attend this accident." Ibid., p. 108.

"When the fractured bones maintain their natural situation, you are under no necessity of extending or replacing the limb, but of applying a proper bandage."—Ibid., p. 108.

To preserve the portions of a fractured bone in proper position: "Two things are chiefly required to this end: 1. To bind it up properly; and, 2. To lay the limb in a convenient posture. The apparatus for securing the situation of the limb, is composed of bandages, bolsters, and splints, which are to be made of thick paper, or wood; or, if the surgeon shall think proper, of thin plates of copper, brass, steel, tin, or lead. But I think the best are those made of wood or paper. The manner of dressing the limb is as follows: In the first place, a roller is to be passed around the fractured limb; upon this are to be placed bolsters, and over these splints, which are to be secured by a tight bandage over all." Ibid., p. 110.

"It is no wonder, therefore, that fractures are ill cured, where the surgeon is ignorant of the proper methods of applying the bandage, or the patient is unruly, and will not give the limb proper rest." Ibid., p. 111.

"Although great numbers of surgeons, at this time, make it their constant practice, to apply a plaster to the fractured part of the limb before they make the bandage, yet the most prudent and skilful surgeons amongst the moderns entirely reject applications of this kind, as not only useless, but injurious to the patient, for these plasters can do no service without the bandage; but the bandage alone, if it is dexterously made, is sufficient to keep the limb firm; and the plaster carries this inconvenience with it, that it stops up the pores of the skin, and produces tumor and most violent itchings. For my own part, I am entirely of opinion, that all kinds of fractures may be very happily cured without the use of plasters; and I am confirmed in this opinion by long experience." Ibid., p. 111.

"Since the chief help seems to be expected from *bandages,* we should principally contrive, that besides having the general properties of a due length and breadth, they should also be accurately adapted to the shape of the broken limb."

"In order to keep the parts in their natural situation, the bandage should be made pretty firm; but if you tighten it too much, you will interrupt the circulation of the blood, and excite tumors, inflammation and gangrene.—On the other hand, if the bandage is made too loose, it will easily come off, and set the disunited parts at liberty; the middle way, therefore, is most eligible." Ibid., p. 111.

"When the bandage remains sufficiently tight, and no bad symptoms appear, you should not loose it till the fifth or eighth day, but where you have inflammations, tumors, pains, and violent itchings, or where the bandage is too loose or too tight, which is frequently the case, you must instantly take off the dressings and change them.—The second and third dressings must be performed in the same manner with the first, with this only difference, that at the third dressing, if you perceive no tumor, you may make the bandage tighter than before, and by this means prevent the luxurious growth of the callus, which would occasion deformity." Ibid., p. 112.

"If, then, these swellings, and inflammations, and ulcers, be almost peculiar to the legs, and chiefly incident to those who, from their age, or sex. or accidental hurts, may reasonably be supposed to have less firmness either in the textures of their whole bodies, or of this particular part, it should seem a right practice to add an additional strength by bandages and strait stockings: and how safely this may be done, appears from the total vanishing of the tumor by a horizontal posture, without any apparent injury to the health," etc. Heberden's Commentaries, p. 88.

Heberden was born in 1710. The Commentaries were published in 1782.

In regard to the treatment of ulcers, "Enquirer" says: "It is somewhat remarkable, in the history of this subject, that the use of bandages, mentioned by Read, and particularly recommended by Wiseman, appears to have been almost wholly disregarded for many years, till the employment was again suggested by Ney Else, (Medical Obs. and Inq., vol. 4.) This gentleman accidentally observed the advantages of tight bandages, and he availed himself of them in their full extent," etc. Ed. Med. and Surg. Journal, vol. i., p. 188—1805.

"In the list of remedies, firm and well-applied bandages form one of the most important. By compressing the superficial vessels, they render the circulation less languid; and by the support which they give to the muscles and soft parts, they admit of the limbs being often moved without inconvenience; they keep the granulations from rising above the surface, and they dispose the skinning process to take place, by promoting the absorption of neighboring parts; they not only invigorate by general compression, but by the warmth which they retain, and serve for a defence to the new formed parts." Ibid., p. 192. After discussing the relative value of the litharge plaster of Mr. Baynton, the flannel roller, etc., he says:

"Rollers made of strong linen, or calico, appear better adapted for common use."

Henderson says, after speaking of various local applications required by certain circumstances: "and, above all, a flannel roller, properly applied, so as to enable the patient to make use of sufficient exercise, were of great service." Duncan's Commentaries, 1788, vol. iii., p. 297.

I again urge upon the readers of these essays on the bandage, the great necessity they are under to study the whole subject. No amount of study will enable them to know too much, no amount of practice in the application of the bandage will make them more skillful than they should be in its use.

In 1753 the fifth edition of Heister's works was published in England. It was filled with useful instruction on the whole subject of the bandage. From these valuable works, Underwood caught his inspiration on the subject, and his lessons, on the application of the bandage to ulcers, were instructive in an eminent degree. Benjamin Bell followed in the good work, and Mr. Baynton made his useful suggestions on the treatment of ulcers, in 1799. The quotations made above show the estimate placed upon the bandage by those who are considered authorities, and their testimony is entitled to great weight.

The medical profession is deeply indepted to Dr. Baynton for correct views in the treatment of ulcers. He has been sometimes disparaged by "distressed critics," but I doubt whether there has been any material improvement made upon his treatment of the generality of ulcers. In the Medical Gazette, of January 8, 1841, Mr. Joseph Bell, of Barhead, England, reports twelve admirable cases to show the efficiency of Mr. Baynton's method of treating ulcers. A distinguished English medical writer, in refering to them, acknowledges the truth of a suggestion made in a preceding part of this essay. He says: "There is no doubt that the practice is excellent; but the troublesome attention which it constantly requires must always be an impediment to its general and continued use." This is certainly a strange objection to urge against an "excellent practice!"

Mr. Bell, in connection with his twelve cases, says: "To explain at any length the method of this celebrated surgeon (Dr. Baynton) may seem to some superfluous. It is, however, my decided conviction that the majority of practitioners are either ignorant of it, or do not put it into practice. Dr. Hannay, of Glasgow, is the only medical man that I either have seen using it, or heard recommending its employment."

"Baynton's system consists of three parts: first, straps of adhesive plaster, from two to three inches broad, and of sufficient length to surround the limb, and to overlap a few inches. These straps are to be applied by placing the center of the strap exactly opposite the ulcer, over which the free ends are to be crossed, pulling them as tight as the feelings of the patient will permit. The first strap is to be placed a little below the lower margin of the sore; the second is to overlap it a little; the third to overlap the second a little; and so on until the ulcer is completely covered; a soft piece of cloth is then to be placed over the ulcer; but I frequently dispense with this. Secondly, a bandage is to be applied, from the extremity of the limb to the articulation above the sore. Thirdly, this bandage is to be kept constantly wet with cold water. The dressing to be changed every day, or every second day, according to the quantity of discharge and irritability of the ulcer. Such, then, is the Bayntonian system, which, when properly applied, is competent to cure, speedily and effectually, the irritable or inflamed, and the varicose, as well as the indolent or callous ulcer; proper attention, of course, being paid to the improvement of the general health—a *sine qua non* in every plan of treatment."

"In the absence of all practical experience on the matter, the system strikes us as admirably suited for 'irritable ulcers,' which differ chiefly from those of an indolent nature in an inflammatory state of the integuments, with infiltration into the adjoining cellular substance. No better means can be employed to remove these conditions than the bandage and cold water. The pressure removes the inflammation, empties and supports the overloaded and enfeebled capillaries. The cold water is a powerful agent in the removal of the cutaneous inflammation; by decreasing the high temperature of the part, it produces a sedative effect. But these are not the only benefits derived from the bandage and water; they also act as stimulants to the ulcer, and thus establish a new and healthy action in the sore, which is at the same time kept constantly clean. I humbly submit, if this be not as rational a plan to remove the inflammatory condition which surrounds these ulcers, as either leeching or poulticing; and then it has the very great advantage, that the patient can walk about during the time the sore is healing."

I cannot imagine how there can be two opinions on the proper treatment of ulcers. The fact, universally noticed, that the superficial veins of the leg, for want of support are most prone to ulcerative action; while the deep seated vessels, supported by the controlling presence of the muscles, are not liable to that form of inflammation, indubitably points to the necessity of support for the superficial or exposed veins. That support is found in the roller, and its powers may be modified so as to suit almost every condition of ulcerative action. For this reason I have not considered it necessary to be minute or specific in a description of the ulcers, because it is safe to assume that those who have capacity to treat ulcers with the bandage, will have the ability to discriminate the varying conditions of the ulcer, and to modify the use of the roller to suit the different stages of the disease. To illustrate this point, I call attention to a statement made by Mr. Bell, in the quotation made above from his paper. In describing the Bayntonian method of cure, he says: "Thirdly, this bandage is to be kept constantly wet with cold water." This is two sweeping a remark, and would land a mere routinist in a great deal of trouble. There are ulcers that do not need cold water, there are others that require a great deal; some do best with the bandage perfectly dry, others with a slight dampening. And all these conditions may be found in the varying stages of the same ulcer. This illustration shows the necessity of being guided by principle in the use of the bandage, as well as in all other forms of treatment, and may teach the value of a thorough study of the various powers ascribed to the bandage.

I cordially subscribe to the eulogium bestowed by Mr. Bell, on Mr. Baynton's treatment of ulcers. He is occasionally criticised by hobby-riders, but to his practical abilities the profession owes nearly all it knows on the proper treatment of ulcers. His plan is certainly susceptible of some improvement, but he paved the way for the correct appreciation of well regulated mechanical support in the treatment of ulcers.

An objection is sometimes urged to the encircling of the limb with the adhesive plaster; it is said that this acts as a ligature, and may injure the ulcer instead of benefitting it. It seems to me, however, that such results must be from the abuse of the remedies, rather than their proper use. I have not seen anything but benefit from Mr. Baynton's method of treatment, and I have had a long and extensive experience with it. If the proper use of Mr. Baynton's means of cure were open to the objections referred to, it would be singular that the evil effects should not have attracted the attention of the great numbers of surgical writers who have commended Mr. Baynton's method. Mr. Critchett admits that Sir Benjamin Brodie, Liston, Ferguson, Syme, Miller, Chelius, Cooper, Rust, Blandin in the French "Dictionary of Medicine and Surgery," and many others, echo "Baynton's plan," but he thinks their commentaries show some dubiety as to its value. The fact, however, that they relied on the treatment, suggested by Mr. Baynton, shows that they recognised it as the most valuable in their possession. If the "Bayntonian method" were capable, *per se*, of inflicting the injuries referred to by Mr. Critchett, should not something have been heard of them in the twelve cases reported by Mr. Bell, in the "London Medical Gazette?"

Having thus introduced the principle of treatment, laid down in Mr. Baynton's treatise, in 1799, it may not be improper to glance at some of the other forms of treatment, that have been zealously commended.

In Charing-Cross Hospital, London, ulcers are treated with oil of turpentine, administered internally. Mr. Barber, M. R. C. S. E. of Colsterworth has great confidence in the application of common vinegar, by bathing, and the use of lint dipped in vinegar, applied over the ulcer, *and the bandage applied in the usual way.*

Mr. Spry is favorable to the use of Balsam of Peru. Lint is soaked in it, placed upon the ulcer, oiled silk is placed over it, *and a well applied roller over the whole.*

Mr. Chapman says: "After dressing the sore with a compress of wet lint, before the bandage is put on, I encircle the part of the limb on which the ulcer is situated with from three to six, or more, moistened strips of linen or calico, of the same length and breadth as the straps employed by Baynton, drawn tightly, and crossed in precisely the same manner as the adhesive plaster. These wet strips adhere to the skin as closely and smoothly as the strapping; and as each one overlaps the upper third, or half, of that previously applied, they afford an amount of support, or compression, very little, if at all, inferior to it. Over this peculiarity of the water dressing, Mr. Chapman uses the bandage. Of the water dressing, and Mr. Macartney's connection with it, I shall have more to say when I come to the treatment I shall commend for ulcers.

"Dr. Jones, M. R. C. S., paints half an inch around the sore with a strong solution of nitrate of silver, and dresses the sore itself with chalk ointment, spread on lint, and three or four inches above and below, with plaster spread on linen, (composed of equal parts of strong mercurial ointment, yellow wax, and soap cerate, with the addition of one drachm of camphor to each ounce of the compound,) and binds the leg from the toe to the knee with wet cotton bandages, the dressing not to be changed more than once a week. In this way Dr. Jones cured his patients in about two months."

But these specimens of illustrations of the multitudinous methods adopted by surgeons, in the treatment of ulcers, must suffice. The variety is not exhausted, but the space that can be devoted to it is, and I must pass to an examination of some of the recent works on this subject. It is certainly a desirable thing that the profession should agree upon some settled principles of treatment for these affections. Mr. Wakley says, with great truth: "Of no disease does a medical practitioner undertake the management more hopeless of benefit, than a 'bad leg.' Viewed in any light, the malady is associated, in his mind, with tediousness, perpetual trouble, and, in the majority of cases, with unsatisfactory results. This is evidently the consequence of unsettled methods of treatment. The hap-hazard system of practice has had full play in the management of these diseases; for every practitioner has his own mode of treatment, and every nostrum has its day of popularity, only to be thrown aside and replaced by another, the fame of which becomes equally ephemeral. It is not, therefore, surprising that the quacks enjoy so large a share of this kind of practice. John Bell might well say, 'it is impossible to be serious while we enumerate the thousand remedies which have been recommended for ulcers.' "

The disposition which is often manifested by medical men, to hunt up and nurse a little bantling, which they call originality, has been the bane of the profession.—They scorn to be "as other men;" their predominant idea of professional excellence is not connected with the welfare of their patients, but with self-laudation, and they rejoice in making, or claiming changes in the works of experience and observation. This paltry aspiration is the cause of the unsettled condition of many points in the treatment of disease. In tracing this state of things, through various works, I have, however, been forcibly impressed by one prominent fact—in nearly every one of the plans of treatment for ulcers, the bandage plays a conspicuous part, and to its presence, in many of the "plans," is due all the success claimed for some of the adjuncts.

Mr. Walker, who published a treatise, in 1848, on the cure of ulcers by fumigation is, however, an exception to the remark. He dispenses with the bandage, and zealously urges a system of fumigation, with iodine and sulphur. He says his system is very simple, and, by way of illustrating the fact, he remarks that "he has a series of mahogany boxes, having in the cover of each a round aperture for the reception of the limb. Each box communicates below with the engine room of my establishment, whence heated air or warm vapor can be passed into the apparatus. On the bottom of the box is placed a heated iron, and on the latter the powder of iodine and sulphur, which is volatilized by heat." The excessive simplicity of this apparatus must appeal to the general practitioner with more than ordinary force! And if he looks to Mr. Walker's details, and examines the delicate shades of discrimination that have to be adapted to each individual case, he must, if he adopts the treatment, make up his mind to spend his whole professional life, in the treatment of ulcers exclusively. Nothing short of incessant practice could make him an expert operator under Mr. Walker's rules, and no expertness under them could ever make amends for dispensing with the bandage. That agent should be regarded as a *sine qua non* in the treatment of ulcers, no matter what other means are resorted to. What form of pathology that can be that looks to any local application to an ulcer, without the bandage, as a successful treatment, transcends any conception I have of the subject. Mr. Critchett remarks very justly, in allusion to the thousands of vegetable and mineral specifics that are commended, "no application to the wound can ever get rid of the surrounding congestion and morbid deposit, upon which the ulceration depends, and to which it owes its continuance."— There is nothing that can perform the offices of the bandage in the treatment, not only of ulcers, but of many affections that are kindred to ulceration, but which are not usually called by that name. It is the oldest of remedies for such disorders; from the days of Hippocrates and Galen, down to the present, through all the varying schools that have lived and perished, through all the wild, fanciful and chimerical systems and theories that have been in vogue, as well as among the most truthful and observant of sects, the bandage has steadily maintained its position, as, at once, not only the most useful and valuable of all agents for its purposes, but the one of all others, capable of the most general and extensive use. The highest authorities in all ages of medicine have constantly endeavored to bear testimony to its great qualities, and many of the old surgeons had as clear and extended views of its multiform capacity for a great variety of surgical purposes, as any of the most enlightened among the moderns. Why should there, then, be any hesitation about the use of a remedy thus commended? If the accumulated experience of many centuries is of any service, why seek to deviate from that plain path that is studded with monuments of success, and that has all the leading lights of the medical profession playing upon it?

I think that for the most useful adjunct to the mechanical pressure and support of the bandage, in the treatment of various inflammations, the profession is mainly indebted to the invaluable labors of Dr. Macartney, of Dublin. That experienced and excellent surgeon, in his lectures, in the University of Dublin, enforced with great eloquence and power, from the year 1819 to 1836, the uses of the water dressing, and when the bandage and the water dressing are combined in the treatment of inflammations, it is scarcely possible to imagine a want that surgeons can feel in the treatment of those diseases to which either is applicable. But it must be borne in mind that the use of these agents requires judgment; that what is useful in some stages, is not so in all, and that modified conditions of the disease, require modified uses, both of the bandage and of the water dressing. In commenting upon the remark of Mr. Bell, in a former part of this paper, on the subject of "keeping the bandage constantly wet with cold water," I stated that I would recur to this subject again.

If a loose statement of the kind quoted from Mr. Bell, were rigidly followed out in the treatment of ulcers, it would show what could be done, by the abuse of a good remedy. On this point, I prefer using the observations of Mr. Critchett, rather than any of my own. Of the "water dressing," Mr. C. says: "I have tried it extensively; it has the advantages of cleanliness, economy, and simplicity, and is certainly an immense improvement upon the complicated unguents of former days; but it is desirable, if possible, to get at its real value. It answers remarkably well in the transition stage from the sub-acute to the chronic ulcer; but there comes a time when the surface of the sore is either raised or flabby, as in cases where it is combined with entire rest, or becomes dry, and makes no progress towards healing. In either case it has done its work, and some other application must be substituted."

It may be asked, what is the value of various other commendations; such, for instance, as the application of steam to indolent ulcers, so strongly urged by Dr. Macartney. The only answer that I can give to all such queries is, that beyond the adhesive strips, the cold water dressing, and the bandage, I have never found occasion for the use of any local application; in a considerable experience, I have found no form of ulceration that did not yield readily to this treatment—so readily and so well, indeed, that it has hitherto proved unnecessary to seek other agents.

There are reasons, already suggested, for the neglect of these agents in the class of disorders for which they are useful—and they are the labor, and loss of time imposed upon the surgeon. This is an indifferent excuse; and there is another, perhaps, which few would like to acknowledge—the want of skill in the use of the bandage. In relation to the first reasons, I think the supposed economy of labor and time used in ordering poultices, balsams, unguents, &c., compared with that gained in the use of the bandage, is a waste—one involves not a little labor and loss of time, to physician and patient, scattered over a long period; the other involves labor and loss of time within a narrow space, and the patient gains as a consequence of the latter. For, in addition to all the other elements of usefulness that have been allotted to the bandage by the eminent and experienced surgeons of all ages, the practitioner should regard as not the least of its prominent merits, that the patient can take exercise, in many forms of ulceration, and attend to business without materially interfering with the progress of the cure. The value of this consideration is almost inestimable, and it brings up, in its proper place, an investigation of Mr. Critchett's proposed modifications of the Baytonian treatment of ulcers.

I have already expressed my admiration of Mr. Critchett's very excellent treatise on ulceration. The general character of its principles, the soundness and clearness of its pathological views, and the correctness and fulness of many of the original suggestions, constitute Mr. C.'s brochure on ulcers, one of the very best in the English language. It is a source of regret that in the midst of so much that is deserving the warmest and strongest commendation, there should be anything that calls for a different course. But I am persuaded that in proportion to the departure of Mr. Critchett from the general spirit of the Baytonian local treatment, in that proportion has he detracted from the merits of his work. I have already questioned the value of Mr. C.'s animadversions upon Mr. Baynton's peculiar use of the adhesive strips, and have expressed the opinion that the objections were more plausible on paper than real in practice. As an improvement upon Mr. Baynton's method, Mr. Critchett recommends that unglazed calico be spread with the simple emp. plumbi of the Pharmacopia, free from resin. The calico, thus prepared, is to be cut into strips two inches broad, and from twelve to eighteen inches long, according to the size of the limb. These pieces are to be warmed, and placed around the limb, from the toes upwards, somewhat in the style of Scultetus's bandage. In applying the adhesive strip, Mr. C. thinks that the strip should be applied at its middle, and worked around the limb smoothly, until the ends are made to cross each other. These are carried, in this way, from the toes, up the limb to the knee, or even above it in some instances. After the limb is thus enveloped, a calico bandage is to be applied, in order to keep the strips, or straps, as Mr. Critchett calls them, in their place.

That there are conditions of the limbs that would be benefitted by this course, I have no kind of doubt; but as a general application, I more than doubt the value of this practice. Notwithstanding Mr. Critchett's opinion to the contrary, it is difficult to imagine that this course will permit a man to pursue his ordinary avocations. Even the moveable turns of the bandage, and the soft, flexible character of its material, do not always, under all circumstances, permit a man to use his limb freely; what must be the additional constraint, when the whole limb is shingled with adhesive plaster, kept to its place by a bandage? And as repeated applications of cold water, or alcohol and water are essential to the comfort of the patient, under the influence of the bandage, and contribute largely to his rapid recovery, I think in this fact, a reason is found for the rejection of Mr. Critchett's adhesive mechanical support, that far outweighs every possible advantage that any one can conceive it to possess.

Ulcers about the malleolus, or tendo Achilles, when intractable, may be benefitted by Mr. Critchett's plan, but it would not in this case be necessary to carry the straps far up the ankle. Ulcers in all other parts of the leg may be safely entrusted to the combined principles of Mr. Baynton and Dr. Macartney. I have seen all the forms of ulcers that have been described, belonging to the leg, and I have never had any reason to look to any other method of cure, than that comprised in the various modifications of the adhesive strips and bandage of Mr. Baynton, and the water-dressing of Dr. Macartney. But, in any case requiring the extensive use of strips, suggested by Mr. Critchett, I should prefer the plan of Mr. Chapman, referred to in a former part of this paper—the use of wet strips of calico, applied in the way that Mr. Critchett uses his adhesive strips.

Those who may wish to practise the use of the bandage in the cure of disease, must not forget the warnings given in my former paper on this subject. As an encouragement to them to enter upon the study and practice of the bandage, I beg leave to quote the language of Mr. Critchett, in reference to giving mechanical support to a diseased limb: "With regard to the time and labor necessary for the acquisition of a fair amount of dexterity, I admit that it demands some practice and perseverance, and some courage, not to be daunted by early failures; but I can assure my young friends, from personal experience, that it is worth some pains; that practice makes each successive application more easy and rapid; and that when once they have triumphed over the difficulties, they will be most amply rewarded by the success that will attend their labors, and that,.too, in a field where the 'harvest is plenteous and the laborers are few.'"

But there are other considerations in the treatment of ulcers. Invaluable as the local treatment is, as first suggested by Mr. Baynton, there are but few forms of ulceration that do not also require some attention to the constitutional treatment of the patients. The chylopoietic organs require examination, and when disordered, as they generally are, they must be regulated by suitable medicines, and an appropriate diet. The specific ulcers—the strumous, syphilitic, phagedenic, menstrual and malignant—require constitutional treatment, but they all derive great benefit from the local means recommended in this essay, for the simple ulcer. I know of no ulcers, except such as are connected with diseased conditions of the bone, that cannot be cured effectually by the proper use of the means commended in this essay. There are uses of the "water-dressing," but let it not be forgotten that there are also abuses of it; there are uses of the bandage, but it too may be abused. A knowledge of the pathology of ulcers, with skill and judgment in the use of the bandage, water-dressing and adhesive strips, will secure a successful treatment of ulcerations, if the constitution is attended to.

It may not be amiss to refer here to the suggestions of Sir Benjamin Brodie, on the dressing of diseased legs— He says: "When you apply the plaster, it should always be with the heel raised, the patient lying flat on his back, so that the vessels of the leg may be emptied of their blood. The same plan should be adopted when the plaster is taken off. If the leg be hanging down at the time the plaster is applied, the veins are full of blood, and the plaster becomes too loose as soon as the patient puts his leg up."

The removal and renewal of the dressings must be regulated by the nature of the case, for no general rule can be given. Where the bandage is used for the double object of mechanical pressure and water-dressing, it should be renewed often, and, speaking from personal experience, I know of nothing more grateful in some forms of inflammation, than the frequent renewal, in the course of the twenty-four hours, of the wet bandage. Sometimes the bandage may remain a week.

There is one other point on which I consider it important to speak, before passing to other matters. I allude to the fear, often expressed, of healing up old ulcers.— That there are circumstances that justify this fear, I have no doubt, but I think the principle is applied too extensively, and often injuriously. I feel glad to find that the experience I have had in the healing of old ulcers is confirmed by the observations of Mr. Critchett. He remarks: "How far is it desirable or safe, in reference to the general health of the patient, to heal ulcers of the leg, more particularly when of long standing? Extensive opportunities of observing cases that have been rapidly healed, and that after having existed for many years —have convinced me that the dangers of healing old ulcers have been very much exaggerated by the old surgeons."

I am well aware that the subject of the treatment of ulceration has not been exhausted in this essay, but think enough has been said to fix the attention of the reader upon the bandage as the prime agent for repairing the destructive effects of ulceration.

There are other forms of formidable disease, in which the bandage exerts a most happy influence, and to one or two of them, I wish to call attention. The first is that serious inflammation called

*Anthrax, or Carbuncle.*—Almost the constant and uniform language of surgical writers on this disease gives rise to the impression that a considerable sloughing of the cellular tissue is as certain a symptom, as the calorific feeling from which the disease derives its name. Sir. Benjamin Brodie, Samuel Cooper, Bromfield, the Dict. des Sciences Med., Physick, Gibson, and others describe a sloughing process as a certain feature of carbuncle. Dr. Gibson ascribes great credit to Dr. Physick for having pointed out the proper time for the application of caustic. The usual treatment is by emollient poultices until openings are made, and then the cautery is to be used at once. This destructive process, of course, greatly protracts the sufferings of the patients, and adds to the seriousness of the case. After the slough has come away, granulations must form, and much time must elapse before the reparative process can be completed.—Are these things necessary? Is sloughing of the cellular tissue an essential feature of carbuncle? Must the cautery be always used in the cure of anthrax? Is it necessary that a patient shall suffer for months with a carbun cle? I am well prepared to answer all these questions in the negative.

In my former paper on the bandage, I stated that there were certain conditions of diseased action that were considered essential pathological phenomena of those diseases, but which were due entirely to the treatment they received, because while present under one form of management, they were altogether unknown in other forms. The remarks I have made on the pathology of anthrax, and the detail of my own case will illustrate the truth of the statement I have just alluded to.

In January, of this year, a small red pimple appeared on my right hand, late in the evening. It was looked upon as too simple a thing to need attention, although it had pain enough in it to keep up a reminiscence of its presence. On the next morning, it had assumed a formidable appearance. There was a considerable tumefaction, embracing that portion of the hand extending from the third joints of the little finger and the one next to it, up to the wrist joint. The skin was hard and brawny, and of a dark crimson color, especially at the centre of the induration. The burning sensation was as severe as though the hand had been dipped in molten lead, the pain was beyond anything I had ever endured, and there was a sense of excessive weight in the hand, constriction, and a stiffness that entirely prevented any useful movements of the fingers.

I had let the time pass for the use of the bandage, for the suffering soon became so great that pressure could not be endured. My general health, which was perfect, apparently, up to the appearance of the anthrax, began to suffer seriously. I was attacked with violent fever, headache, loss of appetite, and was unable to sleep. I resorted to rigid dieting, and the use of the emollient poultice, renewed every two or three hours. In the course of a few days of incessant and extreme suffering, I felt a fluctuation, but the agony of the hand was so intense, that I had to use chloroform locally to deaden the exalted sensibility, in order to use the lancet. I at length plunged a lancet to the bottom of the abscess, and made a large opening in it, but it discharged nothing but a sanious fluid. The pain extended up the arm, and a large, indurated gland, on the inner edge of the deltoid muscle, and another in the axilla, gave painful evidence that the evil influences of the anthrax were radiating towards the chest.

I reapplied, very foolishly, the emollient poultice, and wore it through the afternoon. At eleven o'clock at night, feeling suspicious of great evil, I removed the poultice, and realized my worst fears. The skin was rapidly passing into the first stages of mortification, and more than half of the outer part of the hand, from the second joints of the fingers already spoken of, up to a short space above the wrist joint, was so livid in its appearance, and of such a deep purple, that I scarcely dared to hope that anything could stop the extensive sloughing. I immediately prepared a number of strips of cotton for the fingers, and broader ones for the hand and arm, wet them with cold water, rolled them, enveloped each finger and the thumb in a tight bandage, and then put a very tight one on the hand. I felt immediate relief from my suffering, and, for the first time in five nights, slept soundly. On removing the bandage from the hand, the next day, to tighten it, I found an entire change in the carbuncle. The mortification of the skin was checked, the threatened sloughing of the cellular tissue was gone, and the carbuncle was secreting a copious yellow, thick pus. I continued to use water-dressing and the bandage, and in the course of a few weeks, the hand was entirely cured, without the slightest sloughing of the cellular tissue, and without the use of cautery. Instead of months of suffering, and a long and tedious reparative process by granulation, I recovered from this formidable anthrax in a remarkably short space of time, and with comparatively little suffering. I give this case as an illustration of the value of the bandage; I could detail other cases, in which its power was equally conspicuous.

*Phlebitis of the Humeral Vein.*—A few years ago, Mr. Charles Kastenbine was attacked with a formidable phlebitis, at the bend of the arm, several days after being bled. I found his arm swollen greatly, up to the shoulder, with violent constitutional derangement. There was severe and constant vomiting, great agitation of the system, and the palpable evidences of the rapid approach of the phlebitis towards the region of the heart. Each finger was enveloped in a narrow bandage, and a wet roller was passed from the hand up to the wound of the arm. A compress, made of calico rolled tightly, was laid along the inflamed vein, up towards the axilla, and the roller was carried over this compress.

In the course of from two to three hours, the relief was manifest. The constitutional disturbance ceased, and all the dangerous symptoms disappeared. The bandage was removed next day, and a large quantity of thick, yellow pus was discharged from the wound in the arm. The bandage and compress were renewed, and in the course of two days more, the use of the arm was perfectly restored.

I cannot dwell with much minuteness upon the remainder of the affections that derive more benefit from the bandage, than from all other remedies. In my former paper on this subject, I referred to the immense superiority of the bandage over every other agent for injuries, and some of the diseases of the joints. Its merits will be conspicuous to all who may try its use in such cases. In wounds of the joints, sprains, dislocations, &c., its benefits are of the highest character. Fistulous abscesses, which have long resisted every other form of treatment, seem to feel the happy influences of mechanical pressure almost from the moment of its use. Contusions, and lacerated wounds, of the severest character, that hold out hope of restoration under any treatment, will more certainly, speedily, and completely recover under mechanical pressure than under any other treatment; on these points I speak from a long experience, and from many comparisons of the bandage treatment, with other means. And I feel sure, that in proper dressings of varicose veins with the bandage, will be found the only certain cure of that affection.

I intended to pursue this subject, and enforce the use of the bandage in the treatment of gun-shot wounds, but that subject has been so well handled by the able and philosophical correspondent of this Journal, who writes over the signature of "C.," and the general principle of the treatment, and the usefulness of the bandage for gun-shot wounds, have been so frequently pointed out in my former essay on this subject, that I do not feel that it is necessary to say any more on the subject.

But I hope I may be indulged in the confident expression of the belief, founded on long and well observed testimony, that a careful study of the bandage is worthy the attention of all medical men; and I feel persuaded that he who is without the knowledge of its great capacity and powers, is without the possession of one of the most important elements of success in surgery.

